# ORDER-DR: external validation of severity grading and referable-risk stratification from fundus images

**DOI:** 10.3389/fendo.2026.1923216

**Published:** 2026-08-03

**Authors:** Quanwei Sheng, Qiyu Dong, Shuang Wu, Weijun Liang

**Affiliations:** 1 College of Information Engineering, Changsha Medical University, Changsha, Hunan, China; 2 The Affiliated Changsha Central Hospital, Hengyang Medical School, University of South China, Changsha, Hunan, China

**Keywords:** calibration, deep learning, diabetic retinopathy, external validation, fundus image, ordinal classification, risk stratification

## Abstract

Diabetic retinopathy (DR) is a common microvascular complication of diabetes, and fundus-image deep learning may support scalable screening and risk stratification. However, models trained on a single public dataset can show threshold shift when externally evaluated, limiting direct translation from internal accuracy to clinically interpretable risk. We developed ORDER-DR, a validation-calibrated dual-branch ordinal-risk framework for five-class DR severity grading and referable DR prediction from color fundus photographs. The final prespecified operating model used a high-resolution EfficientNet-B0 branch and a complementary lesion-order-sensitive risk (LORS) EfficientNet-B0 branch; checkpoints and decision thresholds were selected only from APTOS validation predictions. External validation was performed on 1,744 gradable Messidor-2 images. On held-out APTOS test splits, ORDER-DR achieved quadratic weighted kappa (QWK) 0.8960 ± 0.0049, macro-F1 0.6832 ± 0.0279, and accuracy 0.8352 ± 0.0118. On Messidor-2 with validation-calibrated thresholds, ORDER-DR achieved the strongest external ordinal agreement among the evaluated candidate models, with QWK 0.6423 ± 0.0364 and macro-F1 0.4578 ± 0.0332. The LORS branch retained higher external area under the receiver operating characteristic curve (AUROC) and area under the precision–recall curve (AUPRC), indicating a tradeoff between ordinal grade agreement and referable-risk ranking. Reliability analysis showed a higher referable-risk expected calibration error on Messidor-2 than on APTOS test (0.160 ± 0.008 vs. 0.049 ± 0.001). These findings indicate robust external ordinal consistency for ORDER-DR across datasets and show that operational threshold performance, threshold-independent risk ranking, and probability calibration should be evaluated as complementary dimensions. The primary contribution is a locked and reproducible empirical evaluation framework for DR ordinal grading and referable-risk stratification, integrating high-resolution class-balanced discrimination, ordinal-risk modeling, validation-derived threshold calibration, and external error analysis. At the validation-selected referable-risk threshold, Messidor-2 performance showed a high-specificity operating profile; high-sensitivity screening use can be adapted through operating-point selection and calibration for the target clinical setting.

## 1 Introduction

Diabetic retinopathy (DR) is one of the most important microvascular complications of diabetes and remains a major cause of preventable visual impairment. Its clinical relevance is amplified by the global burden of diabetes and by the fact that sight-threatening changes may remain asymptomatic until advanced stages (1). Population-level evidence has shown that DR and vision-threatening DR affect a substantial proportion of people with diabetes, with risk shaped by diabetes duration, glycemic control, and systemic vascular factors (2). Current diabetes-care standards therefore continue to emphasize regular retinal assessment and timely referral pathways for patients at risk of progressive disease (3).

Color fundus photography is central to DR screening because it is non-invasive, comparatively inexpensive, and suitable for large-scale acquisition. The clinical grading task is not merely the detection of any retinal abnormality; it requires assignment of ordered disease severity categories that guide monitoring intervals and referral decisions. International clinical severity scales formalize this progression from no apparent DR to proliferative disease and diabetic macular edema risk (4). Recent evaluations of handheld cameras and AI-assisted grading further indicate that fundus-image workflows are being considered for settings where specialist availability and imaging infrastructure vary substantially (5).

Deep learning has made automated fundus-image interpretation technically feasible. Large validation studies demonstrated that convolutional models can detect referable DR and related ocular diseases with high diagnostic performance in multiethnic or large-scale retinal image cohorts (6, 7). A pivotal autonomous-AI trial also showed that DR screening algorithms can be evaluated in primary-care settings rather than only in curated benchmark environments (8). More recent systematic reviews continue to support the potential of AI-assisted DR screening, while also emphasizing heterogeneity across study designs, populations, imaging devices, and reference standards (9).

For diabetes-associated complication screening, the evidence base is expanding beyond DR detection alone. A recent systematic review of AI with retinal imaging highlighted the broader promise of retinal photographs for microvascular and macrovascular complication assessment, but also noted that many studies remain limited by retrospective design, incomplete external testing, and sparse real-world implementation evidence (10). This point is particularly relevant for type 2 diabetes risk stratification: a model that performs well on a development dataset may still require careful validation before being interpreted as a dependable tool for screening or referral prioritization.

External validation is therefore a central methodological requirement rather than a secondary analysis. Public-data reproduction work has shown that reported DR model performance can change when algorithms are re-evaluated under different dataset and grading conditions (11). Real-world implementation studies similarly show that AI screening systems can vary markedly in sensitivity and specificity across deployment environments (12). Even foundation-model benchmarks for DR grading suggest that discrimination and calibration may diverge when representations are transferred across datasets (13).

Recent retinal foundation models and large retinal datasets further raise the bar for evaluation. RETFound demonstrated that self-supervised retinal representations can improve generalizable disease detection from retinal images (14). Reviews of discriminative, generative, and foundation models in retinal imaging have nevertheless stressed that clinical deployment still lags behind model development (15). New multimodal retinal datasets designed for DR foundation-model benchmarking also underline the need for fine-grained severity labels and external assessment when judging model reliability (16). Accordingly, the revised evaluation includes both conventional convolutional baselines and a post-lock Swin-Tiny transformer comparator, providing a contemporary reference point while keeping the study focused on the locked ORDER-DR validation framework.

Two modeling issues motivate the present study. First, DR severity is naturally ordinal: confusing no DR with proliferative DR is clinically different from confusing adjacent grades. Standard softmax classification can learn useful representations, but its decision rule does not explicitly account for ordered severity. Ordinal neural-network methods were designed to encode rank consistency in ordered labels (17), and recent DR grading studies continue to investigate dual-head or severity-aware designs for this reason (18). Second, referable DR is a binary risk endpoint used in screening, whereas five-class DR grading is a threshold-dependent ordinal decision. A model can rank referable risk well while still requiring recalibration of grade thresholds across datasets.

These considerations also shape how the present work is reported. Prediction-model and medical-imaging AI reporting guidelines emphasize transparent separation of model development, validation, outcome definition, and performance metrics (19, 20). The CLAIM checklist similarly encourages complete reporting of datasets, preprocessing, training, evaluation, and interpretability analyses for AI medical-imaging studies (21). We also considered STARD/STARD-AI-oriented diagnostic-accuracy reporting principles, including clear description of the target condition, index test, reference labels, prespecified thresholds, and applicability limits (22). In this study, we therefore distinguish internal testing from external validation, threshold-dependent grade assignment from threshold-independent risk ranking, and quantitative performance from qualitative visualization.

We developed ORDER-DR, a validation-calibrated dual-branch ordinal-risk framework for DR severity grading and referable-risk stratification. ORDER-DR couples a high-resolution class-balanced EfficientNet-B0 branch with a complementary ordinal-risk branch. It performs probability-level fusion of their class predictions, derives referable DR risk as *P*(grade ≥ 2), and applies ordinal thresholds learned only from internal validation predictions. The primary model was selected for external validation robustness rather than internal-test performance alone.

The contribution centers on a locked external-validation paradigm for ordinal DR grading and referable-risk stratification. ORDER-DR differs from existing ordinal DR models and generic multi-branch systems in the particular locked combination evaluated here: a class-balanced high-resolution branch, a lesion-order-sensitive risk branch, fixed probability-level fusion, validation-derived ordinal thresholds, and external error analysis without Messidor-2 tuning. The contributions are threefold. First, we present ORDER-DR as a compact two-branch ordinal-risk framework for DR severity grading and referable-risk stratification.

Second, we externally validate the model on Messidor-2 and show that it achieves the strongest external QWK and macro-F1 among the evaluated candidate models, including a post-lock Swin-Tiny comparator. Third, we explicitly report the complementarity between external ordinal agreement and referable-risk ranking: ORDER-DR has the strongest external grade-level agreement, whereas the LORS EfficientNet-B0 branch has higher AUROC and AUPRC. A detailed ablation, operating-point, reliability, and error- distribution analysis demonstrates that the components provide complementary behavior within the final framework. This evidence supports external-validation-led model selection and positions ORDER-DR as the evaluated candidate most aligned with the study objective.

## 2 Materials and methods

### 2.1 Study design

This was an empirical machine-learning study using public retinal fundus image datasets. The unit of analysis was a single color fundus photograph, and no demographic, laboratory, treatment, or visit-level clinical variables were used as model inputs. The development dataset was APTOS 2019 Blindness Detection, and the external validation dataset was Messidor-2. The study was designed around two linked outputs: five-class DR severity grading and binary referable-DR risk stratification. Five-class grading used the original ordinal labels from grade 0 to grade 4, whereas referable DR was defined as grade ≥ 2 to reflect a screening-relevant referral threshold. This referable endpoint intentionally focused on grade-based DR severity. Diabetic macular edema, ungradable image referral, media opacity, other retinal disease, and clinician-directed referral indications are separate clinical pathways that can be incorporated through additional labels or workflow modules beyond the present public-data endpoint. Because the publicly used labels and prediction files in this experiment were image-level resources, all primary analyses are reported at the image level; patient-level screening performance was not inferred.

**Table 1** summarizes the task formulation. The same trained models produced both output types: class probabilities were used for five-class grading, and the sum of probabilities for grades 2–4 was used as the referable-risk score. This distinction was important because a model may rank referable DR risk well while still requiring different grade thresholds for stable five-class classification across datasets. The main reporting framework separated model selection, internal test evaluation, external validation, and validation-calibrated thresholding, following the broader reporting logic recommended for prediction models and medical-imaging AI studies (19–21).

**Table 1:** Task definition and analysis outputs.

Component	Definition	Model input	Model output	Evaluation role
Five-class DR grading	Ordered DR severity categories 0, 1, 2, 3, and 4	Single color fundus photograph	Grade probabilities and final grade assignment	Primary ordinal-grading endpoint; evaluated by QWK, macro-F1, and accuracy
Referable-risk stratification	Binary endpoint defined as grade ≥2	Same fundus photograph and grade-probability vector	Risk score P(y≥2) and thresholded referable prediction	Screening-oriented risk endpoint; evaluated by AUROC, AUPRC, sensitivity, specificity, and Brier score
Validation-calibrated grading	Ordinal thresholds learned only from APTOS validation predictions	Expected-grade score from class probabilities	Fixed thresholds applied to APTOS test and Messidor-2	Tests whether internally selected thresholds transfer to an external dataset without using external labels for tuning

### 2.2 Datasets

APTOS 2019 contained 3,662 labeled fundus images and was used for model development, validation-based checkpoint selection, threshold calibration, and held-out internal testing. For each random seed, images were stratified into train, validation, and test splits with a 70/15/15 ratio. Three seeds were used: 20260617, 20260618, and 20260619. The validation split was used only for selecting checkpoints and estimating ordinal or binary thresholds; the APTOS test split was held out until final internal evaluation.

Messidor-2 was used exclusively for external validation. The official Messidor-2 release contains 874 examinations and 1,748 images, with one macula-centered image per eye for each examination; the analyzed external set contained 1,744 gradable images with adjudicated image-level DR grades. The label distribution was 1,017 grade-0 images, 270 grade-1 images, 347 grade-2 images, 75 grade-3 images, and 35 grade-4 images. Messidor-based datasets are widely used in DR screening research (23), and prior public-data reproduction work has used Messidor-2 to assess cross-dataset generalization (11). Messidor-2 therefore provides an appropriate public external benchmark for this study, while prospective multi-device screening cohorts remain the natural next setting for clinical workflow validation. In the present study, Messidor-2 labels were never used for model selection, branch selection, checkpoint selection, fusion-weight tuning, or threshold selection. Because the analysis files used for this public-data experiment did not provide a common patient identifier across both datasets, results were reported at the image level; patient-level aggregation, bilateral-eye clustering, and patient-level confidence intervals require datasets with consistent patient identifiers.

**Table 2** shows how the two datasets were used. The design intentionally separates development and external evaluation: APTOS supplies the internal training and validation signal, whereas Messidor-2 tests whether the resulting model and validation-derived thresholds remain useful under a different image source, class distribution, and grading context. This separation also defines the evidence boundary of the study: the experiment evaluates cross-dataset generalization between two public fundus-image datasets, not prospective clinical deployment.

**Table 2:** Dataset roles, sample sizes, and leakage-control rules.

Dataset	Role	Images	Label distribution	Use in analysis	Leakage-control rule
APTOS 2019	Development and internal testing	3,662	Five DR grades; split stratified within each seed	Training, validation checkpoint selection, validation threshold calibration, and held-out internal test evaluation	Test split not used for training, checkpoint selection, or threshold estimation
Messidor-2	External validation	1,744	Grade 0: 1,017; grade 1: 270; grade 2: 347; grade 3: 75; grade 4: 35	Finalcross-dataset validation of five-class grading and referablerisk stratification	No external labels used for model selection, branch selection, fusion tuning, or threshold estimation

### 2.3 Candidate models and training

Candidate models were designed to separate three methodological choices: backbone architecture, class imbalance handling, and ordinal-risk learning. Standard ResNet18 and EfficientNet-B0 models trained with cross-entropy served as baseline classifiers. Class-balanced variants used the same backbones but modified the training objective to reduce majority-class dominance. LORS variants used an ordinal-risk formulation to encourage predictions consistent with the ordered nature of DR grades. In the implementation, the LORS branch optimized cross-entropy plus expected-grade distance, binary referable-risk loss, and a distance-weighted soft-label distribution term. ResNet and EfficientNet were included because they are established image-classification backbones with different capacity and scaling properties (24, 25). Class-balanced training was motivated by the effective-number weighting principle for imbalanced visual recognition (26), whereas the ordinal-risk branch was motivated by rank-consistent ordinal modeling for ordered categories (17). To provide a contemporary reference point, we also implemented an ImageNet-pretrained Swin-Tiny transformer baseline at the same 384 × 384 input resolution, using the same APTOS splits, checkpoint rule, and Messidor-2 evaluation protocol. The Swin-Tiny results were added after the primary ORDER-DR model was locked and therefore served as an external comparator rather than a model-selection source.

ORDER-DR was then constructed from two high-resolution branches trained at image size 384: a class-balanced EfficientNet-B0 branch and a LORS EfficientNet-B0 branch. These two branches were selected because the former emphasized balanced multi-class discrimination and the latter emphasized ordered risk ranking. ImageNet-pretrained weights were used when available. For each random seed and each candidate model, the best checkpoint was selected only by validation QWK. No APTOS test labels or Messidor-2 labels were used for checkpoint selection.

### 2.4 Image preprocessing and quality handling

All images were converted to RGB, resized to the configured square input resolution, and normalized using ImageNet channel means and standard deviations. To preserve a reproducible public-benchmark pipeline, we did not introduce additional black-border removal, circular retinal-field extraction, optic-disc or macula registration, histogram equalization, illumination-field correction, or left–right eye orientation harmonization. Horizontal flips were used only as training augmentation and were not intended to standardize laterality. APTOS images were analyzed as provided in the public competition files, and Messidor-2 images were analyzed after filtering to the publicly available adjudicated gradable image set. In this study, an ungradable image denotes an image marked as not gradable in the Messidor-2 grading resource; automated image-quality triage and manual quality adjudication are separate workflow components for future deployment studies.

These choices make the experiment reproducible from public files and define the intended scope of the present evaluation. Operational screening workflows would additionally need image-quality triage and referral pathways for acquisition failure, media opacity, poor fixation, incomplete retinal field, non-DR pathology, and DME-specific indications that are outside the five-class DR grade endpoint evaluated here.

The implementation settings are summarized in **Table 3**. These details are reported to make the training and evaluation pipeline reproducible from the public image files and the split seeds.

**Table 3:** Implementation details for candidate branches and ORDER-DR construction.

Component	Setting
Input resolution	Standard candidate models used the configured model input size; the two ORDER-DR branches and the Swin-Tiny comparator used 384×384 RGB images.
Preprocessing	Images were converted to RGB, resized to the target resolution, and normalized with ImageNet mean and standard deviation; no explicit black-border cropping, retinal-field extraction, laterality normalization, or illumination normalization was applied.
Training augmentation	Random horizontal flipping, random rotation up to 10 degrees, and mild color jitter for brightness, contrast, and saturation.
Optimization	AdamW optimizer, learning rate 2×10^−4^ for the 384 branches, weight decay 10^−4^, cosine annealing schedule, mixed-precision training when CUDA was available.
Training duration	Maximum 30 epochs with early stopping patience of 8 epochs; batch size 24 and 6 dataloader workers for the 384 branch runs.
Class imbalance handling	Class-balanced branches used inverse-frequency sample weighting through a weighted random sampler and class-weighted loss.
Checkpoint selection	The checkpoint with the highest APTOS validation QWK was retained for each model and seed.
ORDER-DR fusion	Fixed 0.5*/*0.5 probability-level fusion of the class-balanced EfficientNetB0–384 branch and the LORS EfficientNet-B0–384 branch; the weight was not tuned on APTOS test or Messidor-2.

For an input fundus image *x*, branch *B* with seed *s* outputs logits $z_{B,s,k}(x)$ for DR grade *k* ∈ {0,1,2,3,4}. The corresponding class probability is obtained by the softmax transformation in (Equation 1):

(1) $$p_{B,s}(y=k|x)=\frac{\exp(z_{B,s,k}(x))}{\sum_{j=0}^{4}\exp(z_{B,s,j}(x))}.$$

**Table 4** summarizes the methodological workflow and shows which data split was used at each stage. This table is intended to make the leakage-control logic explicit: training, validation-based selection, internal testing, and external validation were kept as separate steps.

**Table 4:** ORDER-DR modeling workflow and data usage.

Stage	Operation	Data used	Output	Leakage-control rule
Candidate training	Train baseline, class-balanced, and LORS models across three seeds	APTOS train split	Candidate checkpoints and probability outputs	Validation and test labels not used for parameter learning
Checkpoint selection	Select each model checkpoint by validation QWK	APTOS validation split	One checkpoint per model and seed	APTOS test and Messidor-2 labels not used
ORDER-DR construction	Combine the class-balanced EfficientNet-B0–384 branch and LORS EfficientNet-B0–384 branch by fixed probability-level fusion	Predictions from selected branch checkpoints	ORDER-DR five-class probabilities and referable-risk scores	Fusion weight fixed at 0.5/0.5; no external optimization
Threshold calibration	Learn ordinal grade thresholds and referable-risk threshold	APTOS validation predictions	Fixed thresholds for internal and external evaluation	Thresholds estimated before evaluating APTOS test or Messidor-2
Sensitivity-targeted threshold analysis	Select supplementary referable-risk thresholds to target validation sensitivity ≥0.95 and ≥0.99	APTOS validation predictions	Alternative fixed thresholds for reporting specificity, PPV, NPV, and false negatives	Messidor-2 labels not used to choose these thresholds
Final evaluation	Report internal and external performance	APTOS test and Messidor-2	Three-seed mean and standard deviation for all metrics	External labels used only for final performance calculation

**Figure 1:**
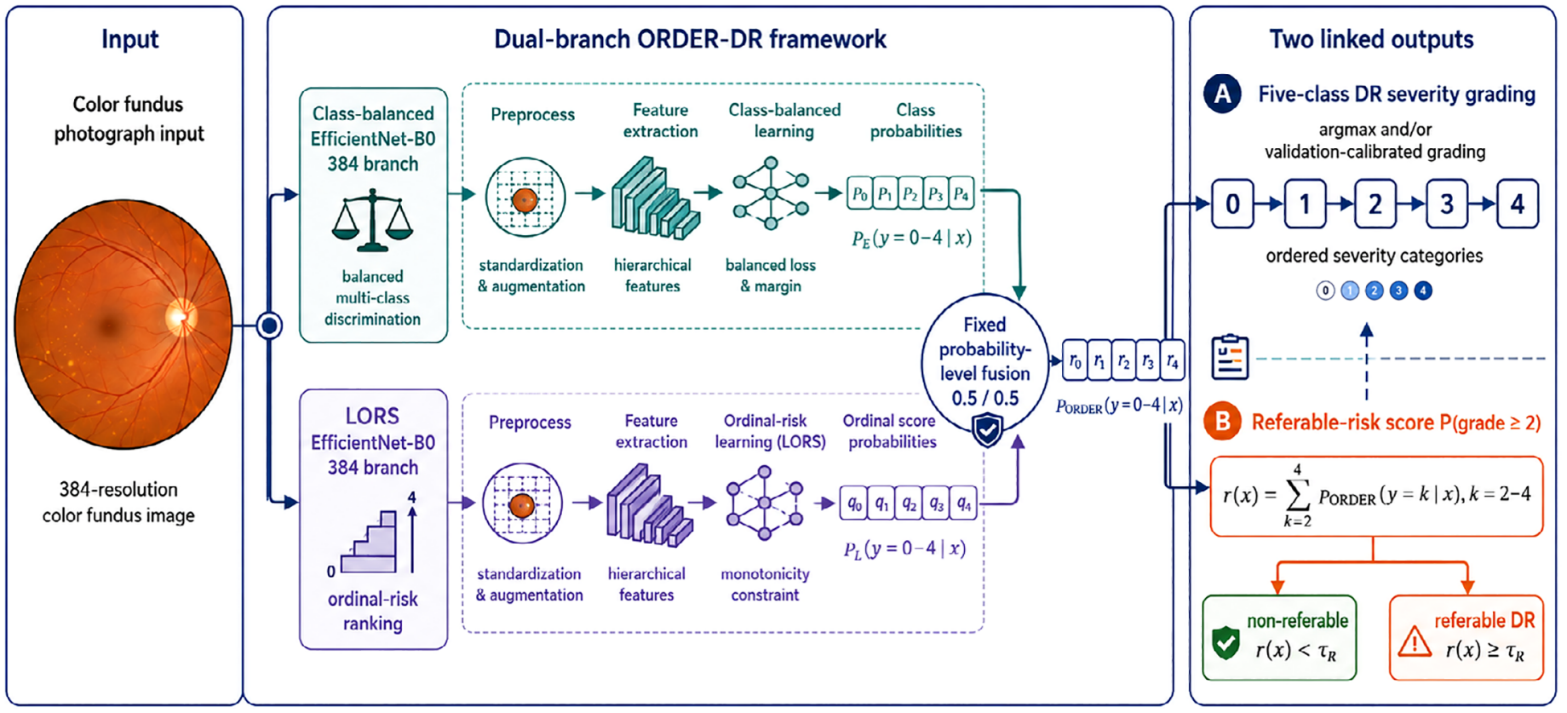
Schematic overview of the ORDER-DR framework. A 384-resolution color fundus image is processed by a class-balanced EfficientNet-B0 branch and a LORS EfficientNet-B0 branch. The selected branch probabilities are combined by fixed probability-level fusion to generate five-class DR severity probabilities and a referable-risk score.

**Figure 1** gives a schematic overview of the ORDER-DR workflow. The figure links the methodological components introduced above to the two study outputs: five-class DR severity grading and referable-risk estimation. Detailed data-use, validation-locking, and threshold rules are provided in **Table 4** and the operating-point tables.

### 2.5 ORDER-DR probability formulation

For seed *s*, let *p_E,s_*(*y* = *k* | *x*) denote the predicted probability for DR grade *k* from the class-balanced EfficientNet-B0 branch, and let *p_L,s_*(*y* = *k* | *x*) denote the probability from the LORS EfficientNet-B0 branch. ORDER-DR uses the fixed probability-level fusion in (Equation 2):

(2) $$p_{\text{ORDER},s}(y=k|x)=\frac{1}{2}p_{E,s}(y=k|x)+\frac{1}{2}p_{L,s}(y=k|x).$$

The default five-class prediction was the argmax grade of the fused probability vector. Referable DR risk was computed as the probability that an image belonged to grade 2 or above, as shown in (Equation 3):

(3) $$r(x)=\sum_{k=2}^{4}p_{\text{ORDER},s}(y=k|x).$$

This formulation allowed the same probability vector to support both study outputs. The full five-class probability distribution was used for ordinal grading, whereas *r*(*x*) in (Equation 3) was used for threshold-independent risk ranking and threshold-dependent referable-risk classification.

### 2.6 Validation-calibrated thresholds

Because external datasets may differ in image acquisition, label distribution, and grading protocols, we evaluated a validation-calibrated ordinal decision rule. For each seed and method, an expected-grade score was computed from class probabilities according to (Equation 4):

(4) $$g(x)=\sum_{k=0}^{4}kp(y=k|x).$$

Four ordinal thresholds *τ*_1_ *< τ*_2_ *< τ*_3_ *< τ*_4_ were selected on the APTOS validation split to optimize QWK. The search used the expected-grade score distribution on the validation split. Candidate threshold values were drawn from score quantiles, initialized at the 20th, 40th, 60th, and 80th percentiles, and then updated coordinate-wise while preserving monotonicity; the retained threshold set was the one with the highest validation QWK. This procedure fixed a deterministic, validation-only ordinal decision rule for each seed. The calibrated grade assignment is defined in (Equation 5):

(5) $${\hat{y}}_{\tau }(x)=\{\begin{array}{ll} 0, & g(x)<\tau_{1},\\ 1, & \tau_{1}\leq g(x)<\tau_{2},\\ 2, & \tau_{2}\leq g(x)<\tau_{3},\\ 3, & \tau_{3}\leq g(x)<\tau_{4},\\ 4, & g(x)\geq \tau_{4}. \end{array}$$

Separately, a referable-risk threshold was selected on the same validation split to optimize binary F1 for referable DR. For binary thresholds, the candidate set included validation risk-score quantiles and fixed reference values. The F1-selected threshold was used as the locked primary operating point. Because screening applications often prioritize sensitivity, two supplementary operating points were also selected on APTOS validation predictions: the highest-specificity threshold satisfying validation sensitivity of at least 0.95, and the highest-specificity threshold satisfying validation sensitivity of at least 0.99. These thresholds were then fixed and applied to APTOS test and Messidor-2. Messidor-2 labels were not used for model selection, branch selection, or threshold selection.

### 2.7 Evaluation metrics

For five-class grading, we reported QWK, macro-F1, and accuracy. QWK was treated as the main ordinal-agreement metric because it penalizes distant grade errors more strongly than adjacent errors. Let *O_ij_*denote the observed confusion matrix, $E_{ij}$the expected matrix under chance agreement, and $W_{ij}={\left(i-j\right)}^{2}/{\left(K-1\right)}^{2}$ for K=5 grades. QWK was computed as (Equation 6):

(6) $$\text{QWK}=1-\frac{\sum_{i=0}^{K-1}\sum_{j=0}^{K-1}W_{ij}O_{ij}}{\sum_{i=0}^{K-1}\sum_{j=0}^{K-1}W_{ij}E_{ij}}.$$

For referable DR, we reported AUROC, AUPRC, sensitivity, specificity, positive predictive value (PPV), negative predictive value (NPV), false-negative counts, and Brier score. AUROC and AUPRC evaluated threshold-independent ranking of *r*(*x*) in (Equation 3); sensitivity, specificity, PPV, NPV, and false negatives evaluated validation-selected operating thresholds; and Brier score quantified probability error for the binary referable endpoint. For five-class grading, we additionally reported grade-specific precision, recall, F1, one-vs-rest accuracy, predicted class distributions, and underestimation counts. Ninety-five percent confidence intervals for grade-specific metrics were calculated from the three prespecified seed-level estimates using a *t* interval and are reported as seed-level descriptive intervals. Referable-risk calibration was summarized with reliability diagrams and expected calibration error (ECE) using ten equal-width risk bins. Paired bootstrap comparisons in the analysis package used 2,000 resamples to quantify uncertainty for selected model contrasts; because only three random seeds were used, these comparisons were treated as descriptive robustness checks. We also generated confusion matrices and per-class recall. The final model-selection decision emphasized three-seed mean performance and external validation consistency. Qualitative interpretability was assessed with Grad-CAM (27) for selected Messidor-2 cases.

## 3 Results

### 3.1 Internal comparison on APTOS

**Table 5** summarizes the full internal comparison on the held-out APTOS test split. The comparison included standard ResNet18 and EfficientNet-B0 baselines, class-balanced training, LORS ordinal-risk training, the two high-resolution branches used by the proposed model, the post-lock Swin-Tiny transformer comparator, and ORDER-DR. The Swin-Tiny comparator achieved the numerically highest internal QWK, macro-F1, AUROC, and AUPRC, confirming that the added modern transformer baseline is a strong reference model. ORDER-DR remained close to Swin-Tiny on internal QWK and macro-F1 while retaining the highest exact five-class accuracy. This indicates that probability-level fusion across the class-balanced and LORS branches improved the final five-class decision relative to its component branches and yielded development-domain performance comparable to a contemporary transformer.

**Table 5:** APTOS held-out test performance for the candidate models.

Method	QWK	Macro-F1	AUROC	AUPRC	Accuracy
ResNet18 CE	0.8896 ± 0.0075	0.6261 ± 0.0150	0.9770 ± 0.0024	0.9569 ± 0.0111	0.8158 ± 0.0052
ResNet18 balanced CE	0.8749 ± 0.0178	0.6491 ± 0.0336	0.9724 ± 0.0048	0.9556 ± 0.0111	0.8048 ± 0.0296
LORS-ResNet18	0.8738 ± 0.0197	0.6221 ± 0.0174	0.9734 ± 0.0023	0.9582 ± 0.0037	0.7891 ± 0.0205
EfficientNet-B0 CE	0.8748 ± 0.0062	0.6397 ± 0.0325	0.9766 ± 0.0059	0.9498 ± 0.0168	0.8073 ± 0.0210
EfficientNet-B0 balanced CE	0.8804 ± 0.0090	0.6407 ± 0.0458	0.9751 ± 0.0022	0.9587 ± 0.0055	0.8055 ± 0.0155
LORS-EfficientNet-B0	0.8845 ± 0.0176	0.6499 ± 0.0393	0.9768 ± 0.0034	0.9544 ± 0.0090	0.8127 ± 0.0293
EfficientNet-B0 384	0.8935 ± 0.0073	0.6660 ± 0.0465	0.9718 ± 0.0048	0.9574 ± 0.0029	0.8212 ± 0.0251
LORS EfficientNet-B0 384	0.8933 ± 0.0042	0.6663 ± 0.0303	0.9781 ± 0.0043	0.9654 ± 0.0045	0.8273 ± 0.0145
Swin-Tiny 384	**0.9074 ± 0.0105**	**0.6918 ± 0.0355**	**0.9812 ± 0.0032**	**0.9701 ± 0.0064**	0.8218 ± 0.0358
**ORDER-DR**	0.8960 ± 0.0049	0.6832 ± 0.0279	0.9759 ± 0.0045	0.9628 ± 0.0036	**0.8352 ± 0.0118**

Values are mean ± standard deviation over three seeds under default argmax prediction. Best values are bolded, and second-best values are underlined.

The gain of ORDER-DR on APTOS was incremental because several baselines were already strong on the internal split. The internal difference between Swin-Tiny and ORDER-DR was small relative to the overall performance level: QWK differed by 0.0114 and macro-F1 by 0.0086, whereas ORDER-DR had higher accuracy by 0.0134. The macro-F1 comparison is especially relevant because the APTOS labels are imbalanced and minority grades can be obscured by accuracy alone. AUROC and AUPRC quantify referable-risk ranking, which is related to but distinct from five-class severity grading; the internal comparison therefore supports evaluating models from the combined perspectives of ordinal agreement, class balance, risk ranking, and exact grade accuracy rather than from a single metric.

### 3.2 External validation on Messidor-2

**Table 6** reports Messidor-2 external validation after thresholds were learned only from APTOS validation predictions. This external evaluation was the most important stress test because the model was trained on APTOS but evaluated on a dataset with different image characteristics, grading distribution, and class imbalance. Under this setting, ORDER-DR produced the strongest external ordinal-grading performance. Compared with the best non-384 baseline, QWK increased from 0.5047 to 0.6423, and macro-F1 increased from 0.3710 to 0.4578. Compared with the strongest high-resolution component, ORDER-DR still improved external QWK from 0.5725 to 0.6423 and macro-F1 from 0.4019 to 0.4578. Compared with Swin-Tiny, ORDER-DR improved external QWK from 0.5628 to 0.6423 and macro-F1 from 0.3599 to 0.4578. Thus, although Swin-Tiny was highly competitive internally, ORDER-DR provided stronger external five-class grading on the primary ordinal endpoints.

**Table 6:** Messidor-2 external validation for the candidate models.

Method	QWK	Macro-F1	AUROC	AUPRC	Accuracy	Brier
ResNet18 CE	0.4389 ± 0.0497	0.3389 ± 0.0365	0.7929 ± 0.0151	0.6348 ± 0.0313	0.6023 ± 0.0235	0.1851 ± 0.0125
ResNet18 balanced CE	0.3505 ± 0.0600	0.2747 ± 0.0154	0.7618 ± 0.0214	0.6165 ± 0.0347	0.5984 ± 0.0116	0.1954 ± 0.0143
LORS-ResNet18	0.3742 ± 0.0438	0.3048 ± 0.0422	0.7546 ± 0.0196	0.5967 ± 0.0329	0.5770 ± 0.0147	0.2019 ± 0.0147
EfficientNet-B0 CE	0.4999 ± 0.0599	0.3486 ± 0.0158	0.8229 ± 0.0052	0.6989 ± 0.0046	0.6005 ± 0.0038	0.1748 ± 0.0092
EfficientNet-B0 balanced CE	0.5047 ± 0.0131	0.3463 ± 0.0615	0.7992 ± 0.0182	0.6764 ± 0.0225	0.5908 ± 0.0381	0.1722 ± 0.0057
LORS-EfficientNet-B0	0.4928 ± 0.0116	0.3710 ± 0.0242	0.8201 ± 0.0097	0.7075 ± 0.0175	0.6070 ± 0.0117	0.1770 ± 0.0070
EfficientNet-B0 384	0.5534 ± 0.0387	0.3931 ± 0.0292	0.8487 ± 0.0108	0.7586 ± 0.0211	0.6273 ± 0.0172	0.1628 ± 0.0066
LORS EfficientNet-B0 384	0.5725 ± 0.0184	0.4019 ± 0.0316	0.8712 ± 0.0272	**0.7837 ± 0.0377**	**0.6414 ± 0.0152**	**0.1570 ± 0.0053**
Swin-Tiny 384	0.5628 ± 0.0255	0.3599 ± 0.0328	**0.8771 ± 0.0062**	0.7491 ± 0.0133	0.6235 ± 0.0075	0.1729 ± 0.0202
**ORDER-DR**	**0.6423 ± 0.0364**	**0.4578 ± 0.0332**	0.8605 ± 0.0158	0.7768 ± 0.0238	0.5214 ± 0.0663	0.1577 ± 0.0053

Values are mean ± standard deviation over three seeds. Ordinal thresholds were learned only from APTOS validation predictions. Best values are bolded, and second-best values are underlined; lower is better for Brier score.

The external results show that ORDER-DR’s main advantage was concentrated in the prespecified five-class severity-grading task. Its Messidor-2 QWK was approximately 0.07 higher than the strongest single branch, and macro-F1 was approximately 0.056 higher, indicating that the dual-branch probability formulation improved both overall ordinal agreement and balanced recognition of minority grades. Swin-Tiny achieved the highest external AUROC, whereas LORS EfficientNet-B0–384 achieved the highest AUPRC and lowest Brier score, suggesting that modern transformer features and ordinal-risk training can both rank binary referable risk strongly. These secondary metric differences do not negate the primary grading result, because ORDER-DR’s external gains over Swin-Tiny were larger for QWK and macro-F1 than Swin-Tiny’s internal gains over ORDER-DR. ORDER-DR also remained second-best for AUPRC and Brier score, with a very small Brier difference from the best model (0.1577 vs. 0.1570). Thus, external validation indicates that ORDER-DR preserved strong referable-risk estimation while providing a larger gain in cross-dataset five-class grading stability. Because the present task combines severity grading and risk stratification, and because five-class ordinal agreement is the core output for differentiating disease stages and supporting stratified management, ORDER-DR was the model most aligned with the study objective. The lower external accuracy of ORDER-DR should be interpreted in this context: validation-calibrated ordinal thresholds can improve distance-weighted agreement and macro-F1 while shifting exact class counts under severe class imbalance.

The locked referable-risk operating point for ORDER-DR is shown in **Table 7**. The referable-risk threshold was learned only on APTOS validation predictions and then applied unchanged to APTOS test and Messidor-2. On Messidor-2, ORDER-DR had a high-specificity operating profile for referable-risk detection. This finding is important for clinical interpretation: the model’s strongest evidence is external ordinal grading consistency, whereas high-sensitivity screening deployment can be configured through prospective operating-point selection according to the intended safety target. At the validation-F1 threshold, the mean Messidor-2 false-negative count was 267.3 ± 12.1 among 457 referable images, showing that this locked high-specificity operating point can be adapted to high-sensitivity autonomous screening through target-specific calibration.

**Table 7:** ORDER-DR referable-DR operating-point performance.

Dataset and rule	Referable AUROC	Referable AUPRC	Brier score	Sensitivity	Specificity
APTOS test, default argmax	0.9759 ± 0.0045	0.9628 ± 0.0036	0.0562 ± 0.0012	0.9043 ± 0.0274	0.9460 ± 0.0098
APTOS test, validation-calibrated threshold	0.9759 ± 0.0045	0.9628 ± 0.0036	0.0562 ± 0.0012	0.9462 ± 0.0119	0.9246 ± 0.0035
Messidor-2, default argmax	0.8605 ± 0.0158	0.7768 ± 0.0238	0.1577 ± 0.0053	0.3494 ± 0.0323	0.9927 ± 0.0040
Messidor-2, validation-calibrated threshold	0.8605 ± 0.0158	0.7768 ± 0.0238	0.1577 ± 0.0053	0.4150 ± 0.0265	0.9855 ± 0.0103

Values are mean ± standard deviation over three seeds. The referable endpoint is grade-based referable DR only and does not include DME, ungradable images, or other clinical referral indications.

**Table 8** reports the supplementary high-sensitivity operating-point analysis. The threshold targeting validation sensitivity ≥ 0.95 produced APTOS test sensitivity 0.9417 ± 0.0323 and specificity 0.9246 ± 0.0179; when transferred unchanged to Messidor-2, sensitivity was 0.4267 ± 0.0616. The threshold targeting validation sensitivity ≥ 0.99 produced APTOS test sensitivity 0.9865 ± 0.0119, specificity 0.8155 ± 0.0798, PPV 0.7895 ± 0.0695, NPV 0.9895 ± 0.0094, and 3.0 ± 2.6 false negatives. When the same threshold was applied unchanged to Messidor-2, sensitivity was 0.6791 ± 0.1183, specificity was 0.8718 ± 0.1285, PPV was 0.7043 ± 0.1674, NPV was 0.8873 ± 0.0256, and the false-negative count was 146.7 ± 54.1. These results show that threshold choice strongly determines the sensitivity–specificity profile and that high-sensitivity referral use should be calibrated in the target dataset rather than transferred solely from APTOS validation predictions.

**Table 8:** Supplementary high-sensitivity referable-DR operating points.

Dataset and validation target	Threshold	Sensitivity	Specificity	PPV	NPV	False negatives
APTOS test, F1-selected threshold	0.267 ± 0.012	0.946 ± 0.012	0.925 ± 0.004	0.895 ± 0.003	0.962 ± 0.008	12.0 ± 2.6
APTOS test, sensitivity ≥0.95	0.242 ± 0.118	0.942 ± 0.032	0.925 ± 0.018	0.896 ± 0.020	0.959 ± 0.021	13.0 ± 7.2
APTOS test, sensitivity ≥0.99	0.014 ± 0.015	0.987 ± 0.012	0.815 ± 0.080	0.789 ± 0.069	0.989 ± 0.009	3.0 ± 2.6
Messidor-2, F1-selected threshold	0.267 ± 0.012	0.415 ± 0.026	0.985 ± 0.010	0.913 ± 0.054	0.826 ± 0.006	267.3 ± 12.1
Messidor-2, sensitivity ≥0.95	0.242 ± 0.118	0.427 ± 0.062	0.982 ± 0.012	0.901 ± 0.057	0.829 ± 0.014	262.0 ± 28.2
Messidor-2, sensitivity ≥0.99	0.014 ± 0.015	0.679 ± 0.118	0.872 ± 0.129	0.704 ± 0.167	0.887 ± 0.026	146.7 ± 54.1

Thresholds were selected only on APTOS validation predictions and applied unchanged to APTOS test and Messidor-2. Values are mean ± standard deviation over three seeds.

### 3.3 Referable-risk calibration

**Figure 2:**
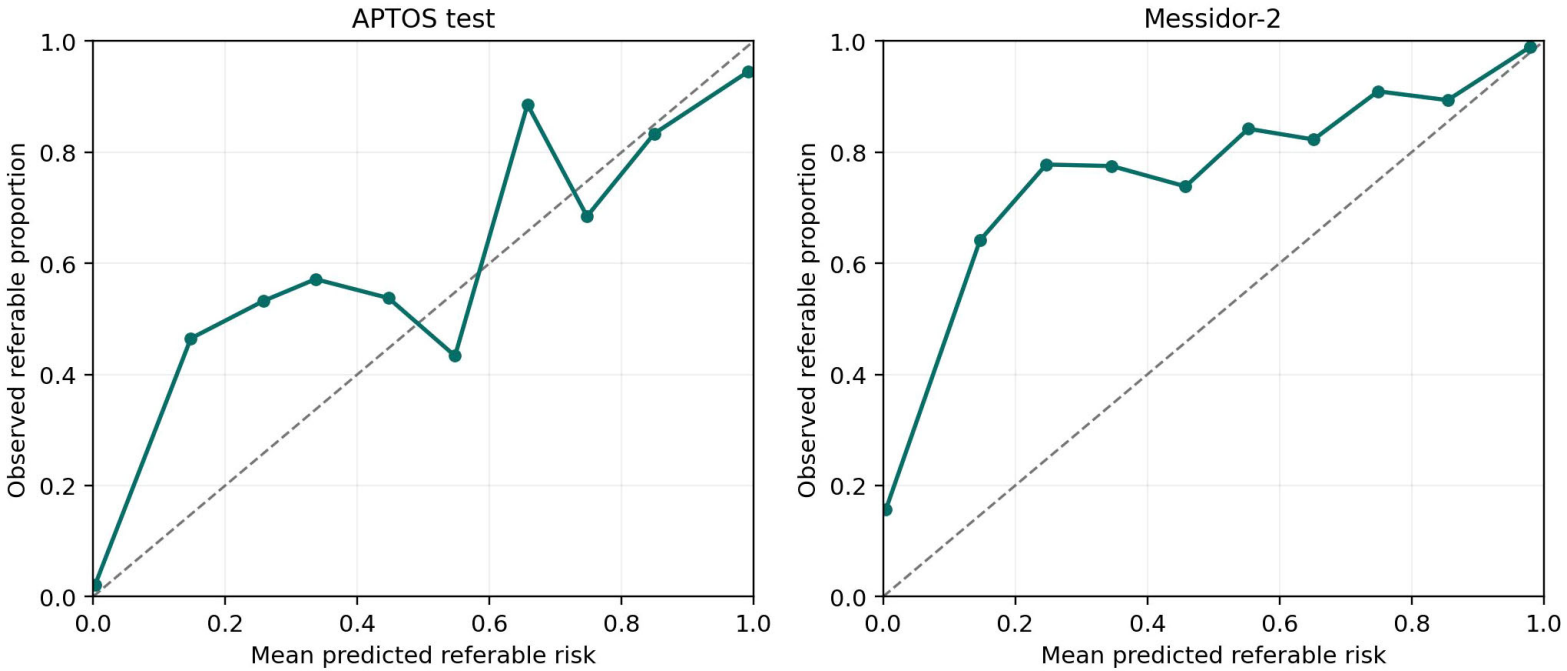
Referable-risk reliability curves for ORDER-DR. Points show ten-bin averages over the three seed-specific reliability summaries. The diagonal line indicates perfect calibration. The Messidor-2 curve and ECE quantify the cross-dataset calibration shift while confirming retained external risk-ranking performance.

**Figure 2** summarizes referable-risk reliability for ORDER-DR. On APTOS test, the Brier score was 0.056 ± 0.001 and ECE was 0.049 ± 0.001. On Messidor-2, the Brier score was 0.158 ± 0.005 and ECE was 0.160 ± 0.008. The reliability curves therefore complement the threshold-sensitivity findings: ORDER-DR retained strong external risk-ranking performance, while absolute probability interpretation in a new screening population can be strengthened by local recalibration.

### 3.4 Component ablation

**Table 9** reports the component ablation of ORDER-DR. This analysis examined whether the proposed model’s performance came from a single dominant branch or from combining complementary branch behavior. Removing the LORS branch left only the class-balanced EfficientNet-B0–384 component; this variant remained competitive internally but had lower external QWK and macro-F1. Removing the class-balanced branch left only the LORS EfficientNet-B0–384 component; this variant produced the best AUROC and AUPRC but did not match the full ORDER-DR framework on external ordinal agreement. The ablation therefore supports the design logic of ORDER-DR: the class-balanced branch contributes stable multi-class discrimination, whereas the LORS branch contributes stronger referable-risk ranking.

**Table 9:** Component ablation of ORDER-DR.

Model variant	APTOS QWK	APTOS Macro-F1	Messidor-2 QWK	Messidor-2 Macro-F1	Messidor-2 AUROC	Messidor-2 AUPRC
Without LORS branch	0.8935 ± 0.0073	0.6660 ± 0.0465	0.5534 ± 0.0387	0.3931 ± 0.0292	0.8487 ± 0.0108	0.7586 ± 0.0211
Without class-balanced branch	0.8933 ± 0.0042	0.6663 ± 0.0303	0.5725 ± 0.0184	0.4019 ± 0.0316	**0.8712 ± 0.0272**	**0.7837 ± 0.0377**
**ORDER-DR**	**0.8960 ± 0.0049**	**0.6832 ± 0.0279**	**0.6423 ± 0.0364**	**0.4578 ± 0.0332**	0.8605 ± 0.0158	0.7768 ± 0.0238

APTOS results use default argmax prediction, whereas Messidor-2 results use validation-calibrated ordinal thresholds. Best values are bolded, and second-best values are underlined.

The full ORDER-DR framework outperformed both ablated variants on APTOS QWK, APTOS macro-F1,Messidor-2 QWK, and Messidor-2 macro-F1. The fact that the complete model did not also dominate AUROC and AUPRC is informative rather than contradictory. It shows that dual-branch probability-level fusion shifted the operating behavior toward more robust five-class grading, while preserving second-best external referable-risk ranking. Thus, the ablation supports ORDER-DR for the manuscript’s primary severity-grading claim, and it also explains why LORS EfficientNet-B0–384 remains clinically relevant when the endpoint is binary referable-risk prioritization.

A *post hoc* fusion-weight sensitivity check was also performed with the available validation-derived alternative fusion rules (**Table 10**). Equal fixed fusion retained the highest external QWK and macro-F1, whereas alternative validation-derived weights produced only small changes in AUROC, AUPRC, and sensitivity. This pattern supports the use of the fixed ORDER-DR formulation for the main severity-grading analysis while showing that the binary referable-risk operating point remains sensitive to threshold and weighting choices.

**Table 10:** Fusion-weight sensitivity check on Messidor-2 external validation.

Fusion rule	QWK	Macro-F1	Referable AUROC	Referable AUPRC	Sensitivity	Specificity
Fixed equal fusion (ORDER-DR)	**0.6423 ± 0.0364**	**0.4578 ± 0.0332**	0.8605 ± 0.0158	0.7768 ± 0.0238	0.4150 ± 0.0265	**0.9855 ± 0.0103**
Validation-QWK weighted fusion	0.6357 ± 0.0416	0.4570 ± 0.0308	0.8579 ± 0.0165	0.7717 ± 0.0288	**0.4420 ± 0.0536**	0.9801 ± 0.0087
Rank-calibrated weighted fusion	0.6396 ± 0.0453	0.4572 ± 0.0351	**0.8617 ± 0.0164**	**0.7778 ± 0.0253**	0.4384 ± 0.0515	0.9816 ± 0.0105

Values are mean ± standard deviation over three seeds using validation-calibrated ordinal thresholds. Best values are bolded, and second-best values are underlined.

### 3.5 Grade-specific external errors and class distributions

**Table 11** gives the Messidor-2 grade-specific results under validation-calibrated ordinal thresholds. Grade-2 recall was 0.189 (95% CI 0.042–0.336), identifying moderate DR as the main target for future threshold and calibration refinement. Grade-3 recall was higher (0.591; 95% CI 0.490–0.692), whereas grade-4 estimates had wider intervals because only 35 grade-4 images were available. These grade-specific results clarify why strong QWK can coexist with lower exact accuracy: the calibrated thresholds reduced severe underestimation and improved ordinal agreement, while exact grade-2 classification remained sensitive to cross-dataset threshold transfer.

**Table 11:** Messidor-2 grade-specific ORDER-DR performance under validation-calibrated ordinal thresholds.

True grade	Support	Precision	Recall	F1	Accuracy
Grade 0	1017	0.777 (0.755–0.800)	0.640 (0.348–0.932)	0.698 (0.512–0.885)	0.684 (0.557–0.810)
Grade 1	270	0.193 (0.169–0.217)	0.505 (0.279–0.731)	0.277 (0.259–0.295)	0.593 (0.429–0.757)
Grade 2	347	0.651 (0.494–0.808)	0.189 (0.042–0.336)	0.292 (0.096–0.487)	0.819 (0.793–0.845)
Grade 3	75	0.572 (0.450–0.694)	0.591 (0.490–0.692)	0.581 (0.472–0.690)	0.963 (0.953–0.974)
Grade 4	35	0.762 (0.306–1.000)	0.333 (0.046–0.620)	0.441 (0.267–0.614)	0.984 (0.979–0.988)

Values are seed-level mean with 95% confidence interval in parentheses, except support. Accuracy is one-vs-rest accuracy for the indicated grade.

The predicted class distribution shifted substantially after applying validation-calibrated ordinal thresholds (**Table 12**). Under default argmax prediction, 85.8% of Messidor-2 images were assigned to grade 0. The calibrated ordinal rule reduced grade-0 assignments to 48.0% and increased grade-1 and grade-3 assignments, improving distance-weighted agreement under the external class distribution. The count of true grade-2 images assigned to grade 0 or 1 changed from 283.0 ± 13.5 to 264.0 ± 13.7, whereas underestimation of grade-3/4 images decreased from 84.7 ± 8.5 to 49.7 ± 6.5. Thus, the QWK gain primarily reflects fewer severe-grade underestimates and smaller ordinal error distances, while the grade-2 decision boundary remains an important calibration target.

**Table 12:** Predicted grade distribution and underestimation summary on Messidor-2 before and after validation-calibrated ordinal thresholds.

Rule	Grade 0	Grade 1	Grade 2	Grade 3	Grade 4
Default argmax, count	1496.7 ± 42.9	81.7 ± 61.3	129.7 ± 25.0	15.7 ± 6.0	20.3 ± 6.4
Default argmax, proportion	85.8 ± 2.5%	4.7 ± 3.5%	7.4 ± 1.4%	0.9 ± 0.3%	1.2 ± 0.4%
Validation-calibrated, count	837.3 ± 151.0	712.7 ± 164.1	99.3 ± 22.7	77.7 ± 3.1	17.0 ± 10.6
Validation-calibrated, proportion	48.0 ± 8.7%	40.9 ± 9.4%	5.7 ± 1.3%	4.5 ± 0.2%	1.0 ± 0.6%
Grade-2 images assigned grade 0–1	283.0±13.5 (argmax)		264.0±13.7 (validation-calibrated)		
Grade-3/4 images underestimated	84.7±8.5 (argmax)		49.7±6.5 (validation-calibrated)		

Values are mean ± standard deviation over three seeds.

### 3.6 Confusion matrices and interpretability

**Figure 3:**
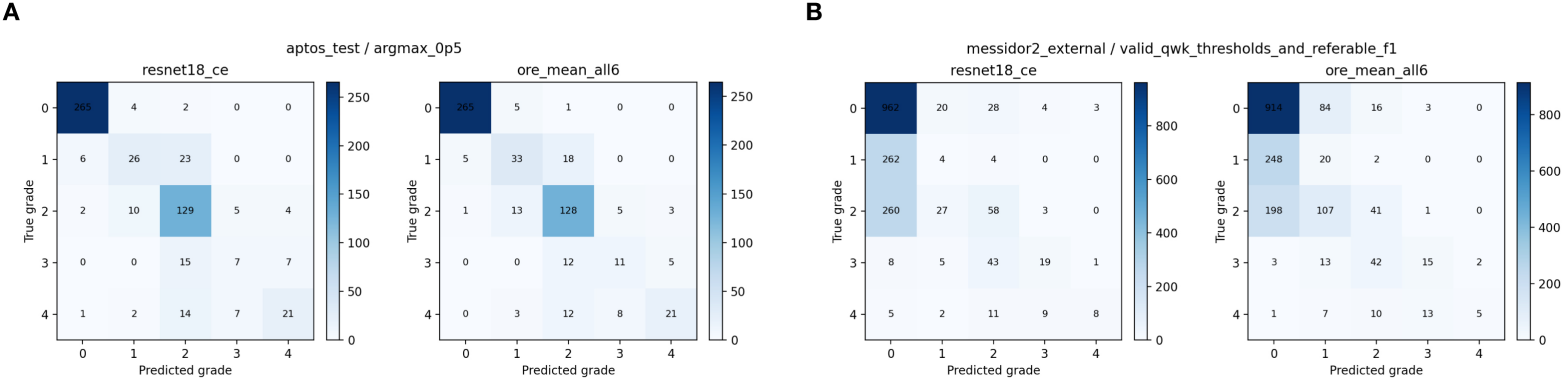
Confusion matrices for internal and external DR severity grading. Values are averaged over three seeds in the corresponding analysis. **(A)** APTOS test, default argmax. **(B)** Messidor-2, validation-calibrated thresholds.

**Figure 3** summarizes representative confusion matrices for internal and external evaluation. On the internal APTOS test split, ORDER-DR separated no-DR images from moderate-to-severe cases relatively clearly, and most errors occurred between adjacent grades, which is consistent with the continuous clinical progression of DR severity. On Messidor-2, despite differences in image source, grade distribution, and annotation characteristics, errors still tended to occur between adjacent or clinically neighboring grades rather than as distant grade jumps. This pattern is consistent with the QWK improvement because QWK penalizes distant ordinal errors more strongly; the external QWK advantage of ORDER-DR therefore indicates that the model reduced not only error frequency but also the ordinal distance of errors.

**Figure 4:**
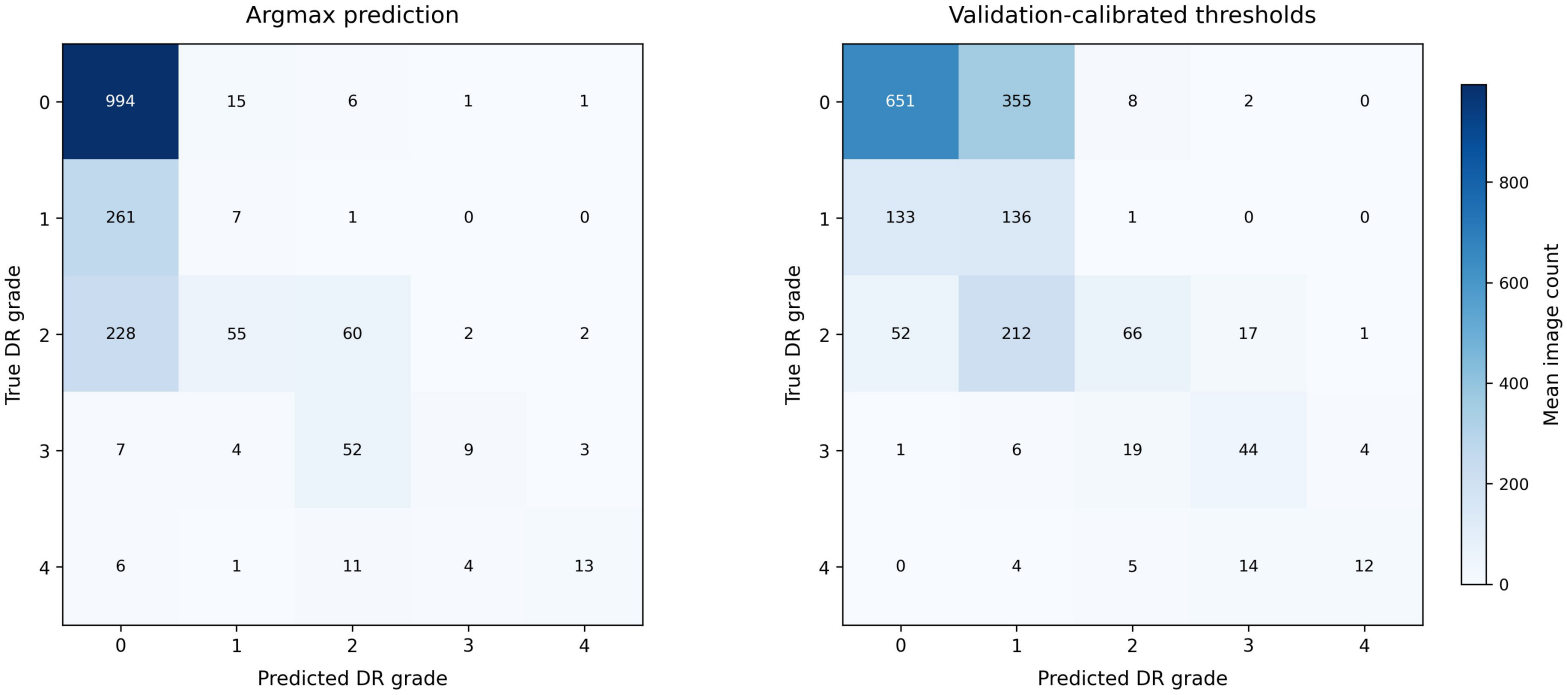
Messidor-2 confusion matrices before and after validation-calibrated ordinal thresholds. The calibrated rule moved many images out of grade 0 and reduced severe-grade underestimation, increasing QWK and macro-F1 while changing the exact-grade distribution for some non-referable and grade-2 images. Values are averaged over three seeds.

The external confusion matrix also shows that severe minority grades remained more difficult to classify exactly. This is compatible with the limited number of grade-3 and grade-4 images in Messidor-2 and with the sensitivity of severe-grade boundaries to cross-dataset threshold calibration. Nevertheless, ORDER-DR maintained the strongest ordinal agreement under validation-calibrated thresholds, supporting its robustness to dataset shift. For clinical interpretation, grade-2 images assigned to grade 0 or 1 represent the key residual error category to monitor when adapting the model for autonomous referral decisions. In a clinician-reviewed triage workflow, adjacent overcalls from grade 0 to grade 1 are less consequential and may be acceptable when the priority is reducing distant ordinal errors. In a risk-stratification workflow, adjacent-grade errors are more compatible with downstream review than distant severity errors, and this error structure is therefore more informative than accuracy alone for evaluating the grading task. **Figure 4** further compares the Messidor-2 confusion matrices before and after applying the validation-calibrated ordinal thresholds.

**Figure 5:**
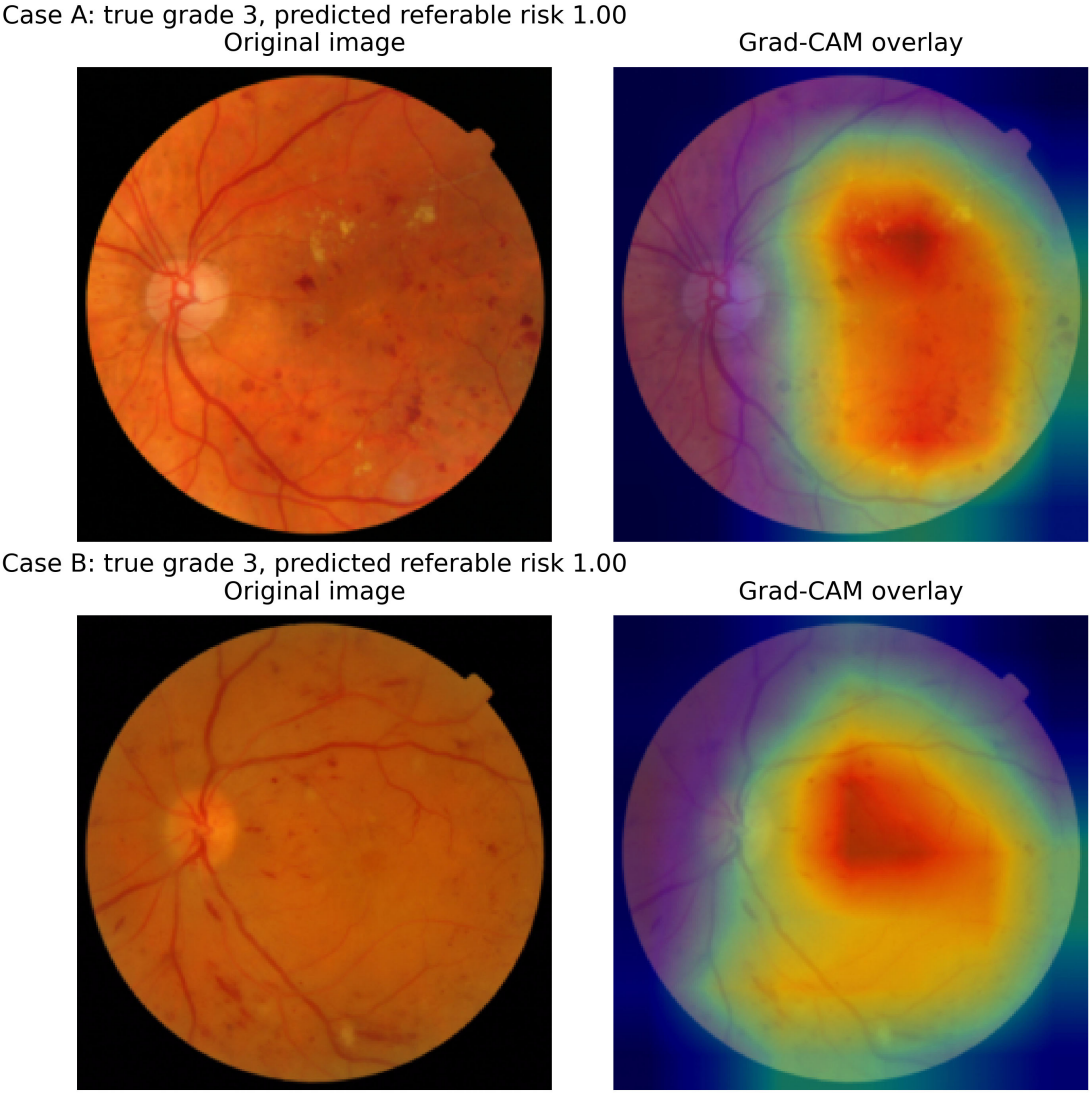
Grad-CAM visualization of representative high-risk true-positive cases from Messidor-2. For each case, the left panel shows the original fundus photograph and the right panel overlays the Grad-CAM activation map for referable DR risk estimated by ORDER-DR. Warmer colors indicate regions contributing more strongly to the model output.

**Figure 5** presents Grad-CAM visualizations for representative high-risk true-positive Messidor-2 images. Both examples were drawn from the external validation set and therefore illustrate the model’s attention pattern under cross-dataset evaluation. The heatmaps show that high-response regions were mainly located over retinal areas with visible abnormality patterns, rather than at image borders, black background, or non-retinal structures. This suggests that ORDER-DR used image content relevant to retinal pathology when estimating high referable-risk probability.

Taken together, the confusion matrices and Grad-CAM examples indicate that ORDER-DR’s advantage was not limited to tabular metric gains. **Figure 1** retains the original framework schematic, while detailed data-use and threshold rules are now shown in **Tables 4**, **7**, **8**. The confusion matrices show that its errors were concentrated near the correct ordinal grade, while Grad-CAM shows interpretable high-risk activation patterns on external images. These two analyses support ORDER-DR as an externally validated model for DR severity grading and referable-risk stratification, and they identify targeted refinement opportunities, including recognition of sparse severe grades and threshold transfer across datasets.

## 4 Discussion

This study developed and evaluated ORDER-DR, a dual-branch ordinal-risk framework for DR severity grading and referable-risk stratification from fundus photographs. Rather than selecting a model solely by internal test performance, the analysis emphasized external ordinal agreement and risk-stratification stability. On APTOS, ORDER-DR remained close to the Swin-Tiny transformer comparator on QWK and macro-F1 while achieving the highest exact five-class accuracy, indicating that probability-level fusion of the class-balanced and LORS branches improved the final grade decision. On Messidor-2, ORDER-DR achieved the strongest external QWK and macro-F1, suggesting more stable modeling of the ordered DR severity structure under dataset shift.

This result has direct task-level implications. DR screening requires not only identifying whether an eye has reached referable severity, but also distinguishing severity grades that may influence follow-up interval, referral priority, and clinical resource allocation. AUROC and AUPRC are well suited for binary referable-risk ranking, whereas QWK and macro-F1 more directly evaluate five-class severity agreement and class-balanced grading. ORDER-DR ranked second for external AUROC and AUPRC while ranking first for QWK and macro-F1, indicating a favorable balance between risk-ranking ability and grade-level agreement among the evaluated candidates. At the locked validation-derived referable threshold, Messidor-2 specificity was high and sensitivity followed a high-specificity profile. The supplementary thresholds selected to target validation sensitivity of at least 0.95 or 0.99 on APTOS changed the Messidor-2 sensitivity–specificity balance, and the reliability analysis quantified external probability-calibration shift. Therefore, the present evidence most strongly supports ORDER-DR as a cross-dataset ordinal grading and referable-risk stratification model. A high-sensitivity clinical screening use case can be implemented through operating-point selection against prespecified sensitivity and specificity requirements, with local recalibration if the intended endpoint is referral triage.

The performance pattern also has a plausible methodological explanation. The class-balanced branch mitigates majority-class bias, whereas the LORS branch encourages learning of the ordinal progression of severity. Fixed probability-level fusion avoids an additional *post-hoc* search over branch weights and reduces the risk of overfitting fusion parameters to the validation split. The ablation results show that the full model outperformed either branch alone on the internal and external grading endpoints, supporting the interpretation that ORDER-DR benefits from complementary branch behavior. At the same time, the strong AUROC and AUPRC of the LORS branch explain why it remains valuable within the framework: it contributes risk-ranking information, while the class-balanced branch helps stabilize five-class decision boundaries. Because the fusion weight was fixed rather than optimized, the study evaluates a deliberately simple probability-level combination rather than a fully searched model-selection procedure. The contribution is therefore the locked model construction, ablation evidence, calibration analysis, and external error characterization of these components in a reproducible public-data setting.

The confusion matrices and Grad-CAM results add context beyond tabular metrics. The predominance of adjacent-grade errors is consistent with the biological continuity of DR progression and with ORDER-DR’s QWK advantage. The threshold comparison further shows how the validation-calibrated ordinal rule improved distance-weighted agreement by shifting the external prediction distribution and reducing severe-grade underestimation, while exact grade-2 recovery remains a calibration target. The Grad-CAM examples show high-risk activation patterns over visible retinal abnormalities in external images, suggesting that the model’s high-risk predictions were linked to retinal image content rather than obvious non-retinal artifacts. For medical AI evaluation, such qualitative evidence does not replace quantitative validation, but it helps interpret model behavior and provides a basis for future integration with clinician review.

The external performance change from APTOS to Messidor-2 is also clinically informative. Messidor-2 was dominated by grade-0 images and contained few grade-3 and grade-4 cases, while differing from APTOS in image source, acquisition context, and grading distribution. These factors can shift both ordinal thresholds and referable-risk calibration. The external Brier score, ECE, and validation-derived operating-point behavior indicate that raw probability output should be locally calibrated before being interpreted as an absolute referral probability in a new population. For clinical decision support, the probability scale and final threshold should be chosen according to the safety requirements of the intended workflow. In a practical referral pathway, ORDER-DR is well suited to clinician-reviewed prioritization of externally acquired images. Under that use case, high-risk cases could be expedited, while low-risk predictions would be interpreted together with image-quality checks and locally validated safety thresholds.

The findings should also be read in light of the study setting. APTOS and Messidor-2 are public datasets that support reproducible model development and external validation, but their acquisition devices, image quality, disease spectrum, and grading conventions may differ from those of prospective screening programs. Messidor-2 is an appropriate and widely used external benchmark for public-data DR research; prospective screening cohorts would further test generalization across cameras, operators, ethnic composition, comorbid ocular disease, media opacity, and failed acquisitions encountered in clinical services. The current analysis used image-level labels and did not estimate patient-level or eye-pair-clustered performance; therefore, the results should not be interpreted as patient-level screening accuracy. Messidor-2 also contains relatively few severe-grade images, which makes severe-grade boundary estimation sensitive to sample distribution.

The present endpoint was restricted to gradable DR severity grades; ungradable-image referral, DME, non-DR retinal disease, retinal-field extraction, laterality normalization, illumination correction, and automated image-quality triage are complementary modules for a complete screening pathway. The use of validation-calibrated thresholds avoided tuning on external test labels, although threshold transfer across institutions and devices warrants larger-scale assessment. In addition, this experiment did not include a same-dataset human-reader comparator, so the absolute clinical acceptability of the observed QWK cannot be inferred from these results alone. Representative misclassified images were inspected qualitatively for Grad-CAM visualization. Future masked retina-specialist review could further classify errors into model behavior, reference-label disagreement, image quality, or competing retinal disease.

Computationally, ORDER-DR uses two EfficientNet-B0 384-resolution forward passes followed by simple probability averaging and thresholding; deployment studies should measure throughput on the target screening hardware to complement the present reproducibility-focused analysis. Privacy-preserving and federated DR studies illustrate one possible route for broader multi-institutional evaluation when direct data pooling is difficult (28). Finally, Grad-CAM was used to examine the relationship between model attention and visible image abnormalities; future studies with more detailed retinal annotations could further characterize how activation patterns correspond to specific lesions.

## 5 Conclusion

This study developed ORDER-DR as a compact dual-branch ordinal-risk framework for fundus-image-based DR severity grading and referable-risk stratification. Across the evaluated candidate models, ORDER-DR achieved stable internal performance on APTOS and the strongest external five-class ordinal agreement on Messidor-2. Compared with single-branch models, ORDER-DR used complementary information from a class-balanced branch and an ordinal-risk branch to improve cross-dataset QWK and macro-F1 while retaining strong referable-risk ranking. These results indicate that explicitly combining class-balanced discrimination with ordinal-risk modeling can improve the robustness of DR grade assignment under dataset shift. For high-sensitivity autonomous screening, ORDER-DR can be paired with target-setting calibration, image-quality triage, and referral criteria that include non-grade indications; these deployment-layer components extend beyond the externally validated grading and risk-stratification endpoint evaluated here.

The findings are relevant to type 2 diabetes complication risk stratification because DR screening requires both binary identification of referable cases and finer differentiation of disease severity. ORDER-DR was not simply optimized for a single internal benchmark; rather, its model selection and interpretation emphasized external validation, validation-calibrated thresholds, and the distinction between grade agreement and risk ranking. Confusion matrix analysis showed that residual errors were concentrated near adjacent grades, and Grad-CAM visualizations indicated that high-risk predictions were associated with interpretable retinal image regions. Together, these findings support ORDER-DR as an externally validated modeling strategy for DR severity grading and risk stratification, while also motivating broader evaluation across additional centers, imaging devices, patient-level samples, and prospective screening workflows to characterize generalizability, threshold stability, and clinical workflow compatibility.

## Data Availability

Publicly available datasets were analyzed in this study. The APTOS 2019 Blindness Detection dataset is available from Kaggle: https://www.kaggle.com/competitions/aptos2019-blindness-detection/data. The Messidor-2 dataset is available from ADCIS: https://www.adcis.net/en/third-party/messidor2/. Analysis code, data splits, prediction files, and result summaries are available at https://github.com/Hajimi-Sudo/ORDER-DR.

## References

[1] Cheung N Mitchell P Wong TY . Diabetic retinopathy. Lancet. (2010) 376:124–36. doi: 10.1016/S0140-6736(09)62124-3 20580421

[2] Yau JWY Rogers SL Kawasaki R Lamoureux EL Kowalski JW Bek T . Global prevalence and major risk factors of diabetic retinopathy. Diabetes Care. (2012) 35:556–64. doi: 10.2337/dc11-1909 22301125 PMC3322721

[3] American Diabetes Association Professional Practice Committee . 12. retinopathy, neuropathy, and foot care: Standards of care in diabetes–2024. Diabetes Care. (2024) 47:S231–43. doi: 10.2337/dc24-S012 38078577 PMC10725803

[4] Wilkinson CP Ferris FL Klein RE Lee PP Agardh CD Davis M . Proposed international clinical diabetic retinopathy and diabetic macular edema disease severity scales. Ophthalmology. (2003) 110:1677–82. doi: 10.1016/S0161-6420(03)00475-5 13129861

[5] Tomic M Vrabec R Hendelja D Kolaric V Bulum T Rahelic D . Diagnostic accuracy of hand-held fundus camera and artificial intelligence in diabetic retinopathy screening. Biomedicines. (2024) 12:34. doi: 10.3390/biomedicines12010034 38255141 PMC10813433

[6] Gulshan V Peng L Coram M Stumpe MC Wu D Narayanaswamy A . Development and validation of a deep learning algorithm for detection of diabetic retinopathy in retinal fundus photographs. JAMA. (2016) 316:2402–10. doi: 10.1001/jama.2016.17216 27898976

[7] Ting DSW Cheung CY-L Lim G Tan GSW Quang ND Gan A . Development and validation of a deep learning system for diabetic retinopathy and related eye diseases using retinal images from multiethnic populations with diabetes. JAMA. (2017) 318:2211–23. doi: 10.1001/jama.2017.18152 29234807 PMC5820739

[8] Abramoff MD Lavin PT Birch M Shah N Folk JC . Pivotal trial of an autonomous 531 ai-based diagnostic system for detection of diabetic retinopathy in primary care offices. NPJ Digital Med. (2018) 1:39. doi: 10.1038/s41746-018-0040-6 31304320 PMC6550188

[9] Tahir H Ullah N Tahir M Domnic I Prabhakar R Meerasa S . Artificial intelligence versus manual screening for the detection of diabetic retinopathy: a comparative systematic review and meta-analysis. Front Med. (2025) 12:1519768. doi: 10.3389/fmed.2025.1519768 40400628 PMC12092458

[10] Yang Q Bee Y Lim CC Sabanayagam C Cheung CY-L Wong TY . Use of artificial intelligence with retinal imaging in screening for diabetes-associated complications: systematic review. EClinicalMedicine. (2025) 81:103089. doi: 10.1016/j.eclinm.2025.103089 40052065 PMC11883405

[11] Voets M Mollersten K Bongo LA . Reproduction study using public data of: Development and validation of a deep learning algorithm for detection of diabetic retinopathy in retinal fundus photographs. PLoS One. (2019) 14:e0217541. doi: 10.1371/journal.pone.0217541 31170223 PMC6553744

[12] Duggal M Chauhan A Gupta V Kankaria A Budhija D Verma P . Real-world evaluation of ai-driven diabetic retinopathy screening in public health settings: validation and implementation study. JMIR Med Inf. (2025) 13:e67529. doi: 10.2196/67529 40925861 PMC12419978

[13] Poyrazer M Yagci H Erten R . How well do frozen foundation models transfer? a calibration-focused benchmark for diabetic retinopathy grading. Front Med. (2026) 13:1815982. doi: 10.3389/fmed.2026.1815982 42078464 PMC13128382

[14] Zhou Y Chia MA Wagner SK Ayhan MS Williamson DJ Struyven RR . A foundation model for generalizable disease detection from retinal images. Nature. (2023) 622:156–63. doi: 10.1038/s41586-023-06555-x 37704728 PMC10550819

[15] Ruamviboonsuk P Arjkongharn N Vongsa N Pakaymaskul P Kaothanthong N . Discriminative, generative artificial intelligence, and foundation models in retina imaging. Taiwan J Ophthalmol. (2024) 14:473–85. doi: 10.4103/tjo.TJO-D-24-00064 39803410 PMC11717344

[16] Tang Z Wang L Guo Z Liang Q Xue S Feng C . A multimodal retinal image dataset for diabetic retinopathy detection using foundation models. Sci Data. (2026) 13:1–13. doi: 10.1038/s41597-026-07005-9 41807442 PMC13102964

[17] Cao W Mirjalili V Raschka S . Rank consistent ordinal regression for neural networks with application to age estimation. Pattern Recognit Lett. (2020) 140:325–31. doi: 10.1016/j.patrec.2020.11.008 38826717

[18] Yu W Si X Zhong J . Dual-swinord: A dual-head swin transformer with semantic prior injection for ordinal diabetic retinopathy grading. Bioengineering. (2026) 13:374. doi: 10.3390/bioengineering13040374 42072168 PMC13112979

[19] Collins GS Reitsma JB Altman DG Moons KGM . Transparent reporting of a multivariable prediction model for individual prognosis or diagnosis (tripod): the tripod statement. Ann Internal Med. (2015) 162:55–63. doi: 10.7326/M14-0697 25560714

[20] Collins GS Moons KGM Dhiman P Riley RD Beam AL Van Calster B . TRIPOD+AI statement: updated guidance for reporting clinical prediction models that use regression or machine learning methods. BMJ. (2024) 385:e078378. doi: 10.1136/bmj-2023-078378 38626948 PMC11019967

[21] Mongan J Moy L Kahn CE . Checklist for artificial intelligence in medical imaging (claim): a guide for authors and reviewers. Radiology: Artif Intell. (2020) 2:e200029. doi: 10.1148/ryai.2020200029 33937821 PMC8017414

[22] Bossuyt PM Reitsma JB Bruns DE Gatsonis CA Glasziou PP Irwig L . STARD 2015: An updated list of essential items for reporting diagnostic accuracy studies. BMJ. (2015) 351:h5527. doi: 10.1136/bmj.h5527 26511519 PMC4623764

[23] Decenciere E Zhang X Cazuguel G Lay B Cochener B Trone C . Feedback on a publicly distributed image database: the messidor database. Image Anal Stereol. (2014) 33:231–4. doi: 10.5566/ias.1155

[24] He K Zhang X Ren S Sun J . (2016). “ Deep residual learning for image recognition”, in: Proceedings of the IEEE Conference on Computer Vision and Pattern Recognition, Las Vegas, NV, USA: IEEE. 770–8. doi: 10.1109/CVPR.2016.90

[25] Tan M Le QV . (2019). “ Efficientnet: Rethinking model scaling for convolutional neural networks”, in: Proceedings of the 36th International Conference on Machine Learning, Long Beach, California, USA: Proceedings of Machine Learning Research (PMLR). 6105–14.

[26] Cui Y Jia M Lin T-Y Song Y Belongie S . (2019). “ Class-balanced loss based on effective number of samples”, in: Proceedings of the IEEE/CVF Conference on Computer Vision and Pattern Recognition, :Long Beach, CA, USA IEEE. 9268–77. doi: 10.1109/CVPR.2019.00949

[27] Selvaraju RR Cogswell M Das A Vedantam R Parikh D Batra D . Grad-cam: Visual explanations from deep networks via gradient-based localization. Int J Comput Vision. (2020) 128:336–59. doi: 10.1007/s11263-019-01228-7 30311153

[28] Nielsen C Wilms M Forkert ND . Highly efficient homomorphic encryption-based federated learning for diabetic retinopathy classification. J Med Imaging. (2025) 12:34504. doi: 10.1117/1.jmi.12.3.034504 40463488 PMC12128631

